# Improving the Ability of a Laser Ultrasonic Wave-Based Detection of Damage on the Curved Surface of a Pipe Using a Deep Learning Technique

**DOI:** 10.3390/s21217105

**Published:** 2021-10-26

**Authors:** Byoungjoon Yu, Kassahun Demissie Tola, Changgil Lee, Seunghee Park

**Affiliations:** 1 Department of Convergence Engineering for Future City, Sungkyunkwan University, Suwon 16419, Korea; mysinmu123@skku.edu; 2 Department of Civil, Architecture and Environmental System Engineering, Sungkyunkwan University, Suwon 16419, Korea; kastolla@skku.edu; 3 Advanced Infrastructure Convergence Research Department, Korea Railroad Research Institute, Uiwang 16105, Korea; tolck81@krri.re.kr; 4 School of Civil, Architectural Engineering and Landscape Architecture, Sungkyunkwan University, Suwon 16419, Korea; 5 Technical Research Center, Smart Inside Co., Ltd., Suwon 16419, Korea

**Keywords:** plumbing maintenance, deep learning, ultrasonic wave propagation imaging, CNN, external damage

## Abstract

With the advent of the Fourth Industrial Revolution, the economic, social, and technological demands for pipe maintenance are increasing due to the aging of the infrastructure caused by the increase in industrial development and the expansion of cities. Owing to this, an automatic pipe damage detection system was built using a laser-scanned pipe’s ultrasonic wave propagation imaging (UWPI) data and conventional neural network (CNN)-based object detection algorithms. The algorithm used in this study was EfficientDet-d0, a CNN-based object detection algorithm which uses the transfer learning method. As a result, the mean average precision (mAP) was measured to be 0.39. The result found was higher than COCO EfficientDet-d0 mAP, which is expected to enable the efficient maintenance of piping used in construction and many industries.

## 1. Introduction

Piping is widely used as an important material not only in construction but also in many industrial fields such as aviation and machinery, and as a result the economic, social and technical demands for maintenance due to aging are increasing. In many industrial fields, it is required to apply inspection technology that can detect pipe damage at an early stage [1].

Through laser scanning-based research conducted in previous studies, the applicability to steel and bolt loosening was confirmed [2,3,4,5,6,7]. On account of this, we aim to detect damage to the pipe with ultrasonic wave propagation imaging (UWPI) in the current work.

The UWPI is one of the signal processing methods that excites a test object with a Q-switched pulse laser system, measures it with an acoustic emission (AE) sensor that acquires the wave propagation data, and displays it as waveform data using an image [8].

Many academic research activities are being carried out on piping. Intensity-based optical system [9,10], microwave nondestructive testing [11], pipe NDT inspection using an automated robot [12,13,14], eddy-current-based crack recognition [15], etc., are among the investigated techniques. However, most damage detection techniques depend on the empirical and subjective judgment of experienced experts. To overcome this problem, a lot of research based on computer vision technology using machine learning [16] is being conducted in the fields of structural health monitoring (SHM) and nondestructive evaluation (NDE) [17].

Recently, research using deep learning technology among various machine learning technologies has been actively conducted. Among the various deep-learning-based technologies, image classification using CNN shows better performance results than existing image classification algorithms and is continuously being researched and developed [18]. To detect pipe damage through CNN-based object detection, a large amount of data is required. In addition, it is difficult to obtain data and a lot of learning time is required. In this regard, we intend to utilize the transfer learning [19] technique that enables efficient learning using a small amount of data. Using the pre-learned COCO 2017 EfficientDet-d0 model, it is proposed to detect a damage in piping by laser scanning utilizing UWPI. The main objectives of this study are as follows. The primary goal of this study is to confirm the possibility of establishing a damage detection system through CNN learning on the ultrasonic wave propagation images found from the laser scanning of a pipe. Next, by applying the transfer learning technique, we want to check whether it is possible to efficiently detect damage with only a small amount of UWPI learning data. The structure of this paper is as follows. Section 2 describes the UWPI system and its theory that utilizes laser scanning technology to create training data. Section 3 describes the CNN algorithm and EfficientDet-d0 model used to detect pipe damage. In Section 4, we present the experiments and experimental results, and in Section 5 we present the conclusion of the study.

## 2. Ultrasonic Wave Generation Mechanism Using Pulsed Laser

### 2.1. Ultrasonic Wave Mode Generation Theory

**Figure 1:**
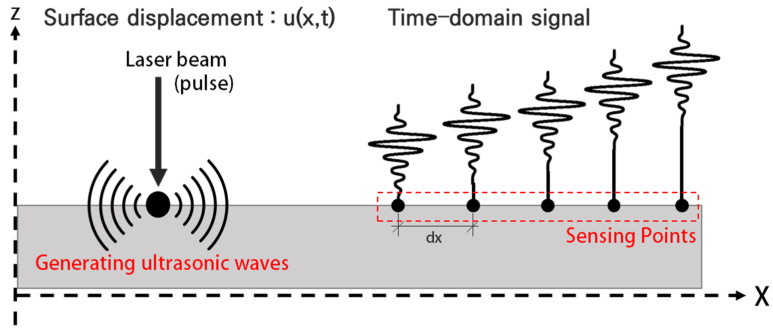
Ultrasonic wave mode generation in a plate [20].

The generation of ultrasonic waves by a pulsed laser and the sensing of the generated waves takes place as shown in Figure 1 [20]. A source of impulsive pressure is applied to the surface and the resulting time records are tracked at different locations on the surface. When a pulsed laser beam collides with a target structure, various physical phenomena can occur. The basic problems of ultrasonic thermoelasticity generation can be divided into three sub-problems: moderate absorption of electromagnetic energy, reflection, transmission of the laser radiation. As a result of these processes, the absorbed laser energy causes local heating of the area, leading to a thermoelastic expansion of the material and the generation of ultrasonic waves [20,21,22].

### 2.2. Ultrasonic Wave Propagation Imaging System Configuration

**Figure 2:**
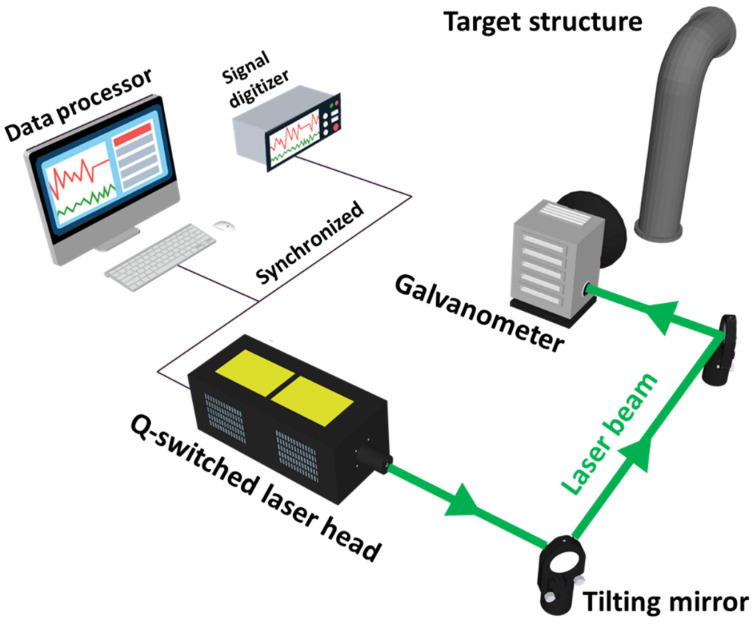
Conceptual diagram of an UWPI system.

The UWPI system consists of a Q-switched laser system, a galvanometer (laser mirror scanner), an AE sensor (ultrasonic sensor), a digitizer and an image processor as shown in Figure 2. All devices are synchronized and the ultrasonic response signal is measured by the AE sensor in the digitizer at the same time.

A galvanometer is used to target a structure with a laser mirror scanner, to specify a point at the desired location and use it for laser pulse incidence. The laser mirror scanner is driven by two tilting mirrors and is designed to operate at a wavelength of 1064 nm. The maximum angular velocity of the galvanometer is 100 rad/s within the range of ±0.35 rad (±20.05°). The rotation axes of the two tilting mirrors are perpendicular to each other, which allows the laser mirror scanner to scan the 2D scan area at high speed. The scanning takes place as follows: the laser mirror scanner first performs an upward scan on the vertical axis, then moves to the horizontal axis to perform a downward scan after the vertical axis scan is complete. Through these scanning processes, ultrasonic waves are arranged on the target structure in the form of a grid. The details of the laser system are as specified in Table 1.

**Figure 3:**
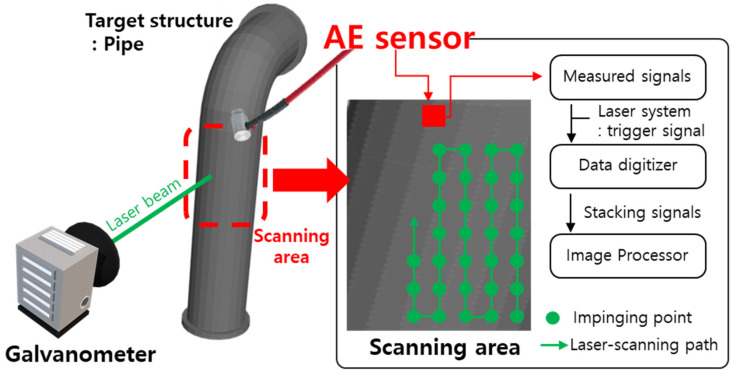
Laser-induced UWPI system.

To drive the UWPI system and acquire the data required for ultrasound images from the acoustic emission sensor (AE sensor) with a built-in amplifier, the UWPI control system was configured as shown in Figure 3 using LabVIEW. The software program consists of a scanning grid configuration for a test object, a parameter setting part (sampling, frequency, number of measured samples, trigger signal level, etc.) necessary for a digitizer, a laser system and laser mirror scanner communication parameter setting part and an ultrasound imaging part.

### 2.3. Ultrasonic Wave Propagation Imaging Algorithm

**Figure 4:**
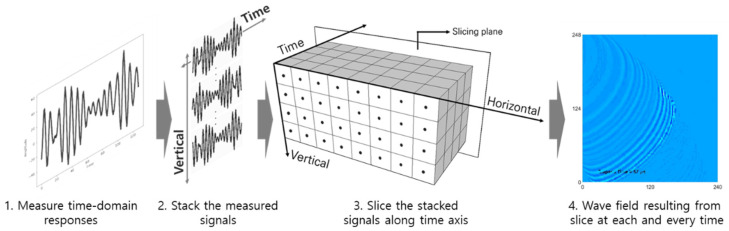
Process of ultrasonic wave propagation imaging (UWPI) system [5].

The steps to generate an ultrasonic wave image using the ultrasonic signal in the time domain measured by the UWPI system consist of a total of three steps as shown in Figure 4. First, the measured time domain signals are arranged on a vertical plane. At this time, each measurement signal is positioned at the laser beam incident point to construct 3D data of the horizontal axis, the vertical axis, and the time axis. The value at each excitation point on this plane becomes the ultrasonic amplitude value at a specific time instant, and if the image is reproduced repeatedly along the measurement time on the time axis and then played in quick succession, an ultrasonic wave propagation movie can be obtained [5].

## 3. Deep Learning-CNN

Deep learning refers to machine learning techniques that construct a model with a large number of neural layers for pattern recognition problems or feature point learning [23]. Since the publication of the deep belief network paper by Hinton at the University of Toronto in Canada in 2009 [24], deep learning has been developed along with various algorithms in many industries [25]. The neural network structures to which deep learning technology is applied include auto-encoders, restricted Boltzmann machines (RBMs), convolutional neural networks (CNNs), and recurrent neural networks (RNNs) [26,27,28,29]. In this study, we intend to utilize a CNN, which has been in the spotlight in image recognition and classification fields, to determine the presence or absence of damage to piping structures through image learning.

### 3.1. CNN

The conventional neural network (CNN) was devised by LeCun of New York University, USA, and it is one type of deep learning. It is the most popular algorithm in the field of image recognition and classification [30]. CNNs have made great strides in image recognition and classification and has shown tremendous performance in computer vision [31].

**Figure 5:**
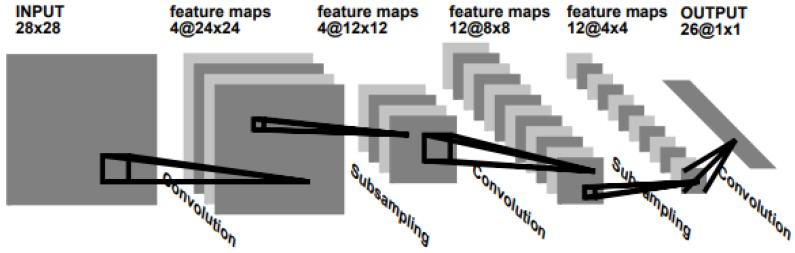
Convolutional neural network (CNN) for image processing [25].

The basic structure of the CNN is shown in Figure 5 below. As indicated, it passes the image through the filter of the convolution layer and the pooling layer repeatedly, and classifies the image through the existing fully connected network, multilayer perceptron and softmax algorithm.

Typically, through TensorFlow [32] and Keras [33], which are open source software provided by Google, people who are not computer developers can use image recognition and classification using deep learning and CNN.

### 3.2. Object Detection

Object detection (OD) refers to an important computer vision task in digital image processing that can detect instances of visual objects of a specific class (human, animal, car, etc.) [34]. Generally, it is divided into general object detection and detection applications. Detection applications refer to applied detection technologies such as COVID-19 mask detection and automatic vehicle number recognition systems that are commonly seen around. In this study, we intend to perform the learning on laser scanning images of the pipe and detect the damage by using application-specific detection.

### 3.3. EfficientDet

**Figure 6:**
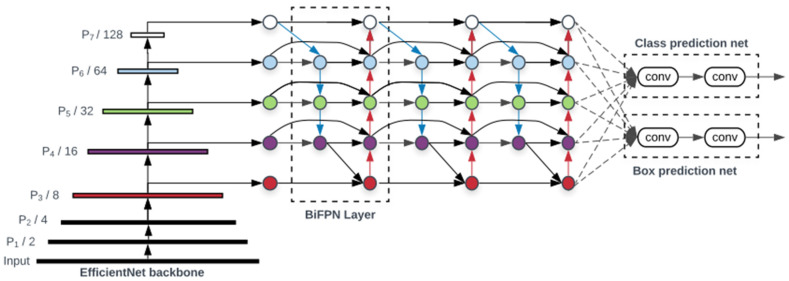
EfficientDet architecture [35].

EfficientDet used in this study ranked first among the models whose performance was measured without extra training data in the 2019 Dataset Object Detection competition on the COCO minival dataset, and it was found that it is an efficient network with good performance, that is, with a low amount of computation (FLOPS) and good accuracy [35]. It is an object detection algorithm that achieved the highest mAP in performance comparison experiments conducted with single-model single-scale and updated SOTA (state-of-the-art, the current highest level of results). Therefore, EfficientDet presents two differences compared with existing models. First, the existing models have developed a cross-scale feature fusion network structure, but EfficientDet pointed out that the contribution to the output feature should be different because each resolution of the input feature is different. To resolve this problem, a weighted bidirectional FPN (BiFPN) [35] structure was proposed as shown in Figure 6. EfficientDet employs EfficientDet [36] as the backbone network, BiFPN as the feature network, and a shared class/box prediction network. Second, the existing models depended on huge backbone networks for large input image size for accuracy, but EfficientDet used compound scaling, a method of increasing the input resolution, depth, and width, which are factors that determine the size and computational amount of the model simultaneously and increase them.

## 4. UWPI-System-Based Pipe Damage Detection Experiment and CNN Learning

### 4.1. Detecting External Damage to Pipe Bends Using UWPI System

**Figure 7:**
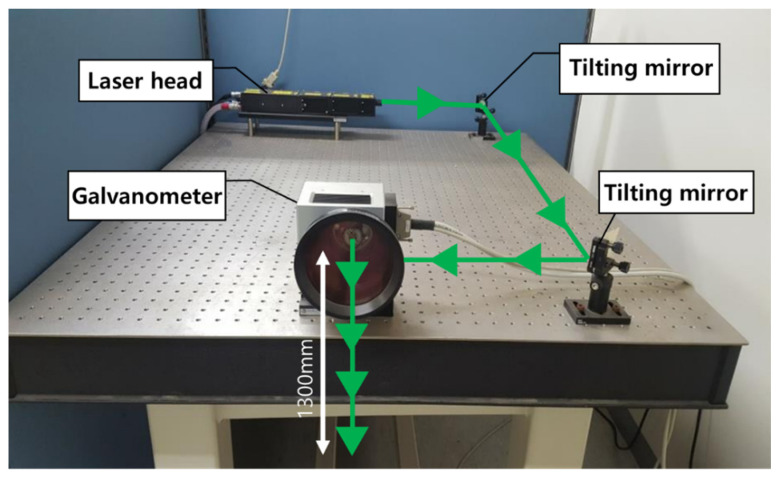
A noncontact laser ultrasonic scanning system composed of a Q-switched Nd:YAG pulsed laser with a galvanometer for ultrasonic excitation scanning [5].

To obtain an image of pipe damage to be used in this study, a Nd:YAG pulse laser was used to generate Lamb waves, and an AE sensor was used to measure the waveform. The laser system used in the experiment is shown in Figure 7.

The Q-switched Nd:YAG pulse laser emits a laser beam through a galvanometer after a trigger signal is delivered [5]. Using the mirror inside the galvanometer, the laser beam is emitted to the target point along the scan path, and the measured data are sent to the digitizer through the acoustic emission (AE) sensor. Then, the digitized signal reaches the image processor, where the UWPI process occurs [6].

**Figure 8:**
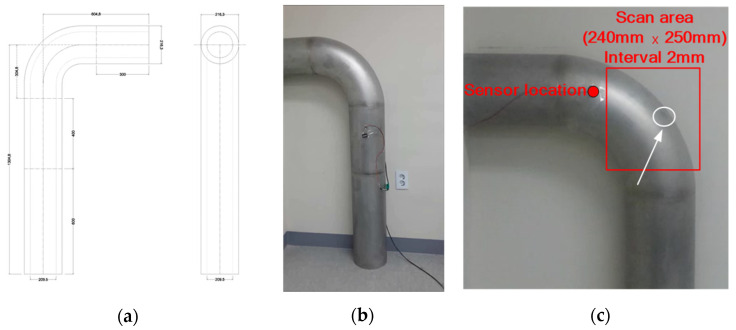
Stainless steel pipe damage diagnosis test specimens. (**a**) Stainless steel 304 specimen drawing; (**b**) stainless steel pipe specimen; (**c**) artificial damage carved on the specimen.

For the test pipe utilized in this study, a stainless steel 304 specimen was used, and on the curved surface of a pipe a 1 mm deep damage was artificially applied to a diameter of 30 mm, as shown in Figure 8.

**Figure 9:**
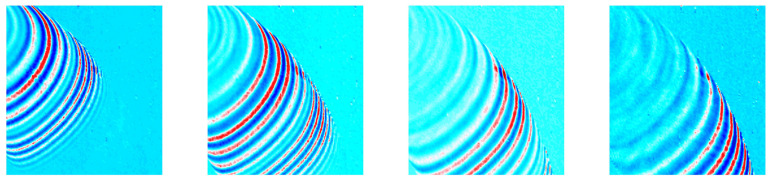
UWPI unfiltered video data to be used for deep learning.

The laser scanning area is 240 mm wide and 250 mm high, and the laser excitation interval is 2 mm. The number of laser excitation points is 15,125, and the scanning time is 12.6 min. The result of the UWPI after scanning the pipe bend is shown in Figure 9.

### 4.2. CNN Learning Using Damage Data

**Figure 10:**
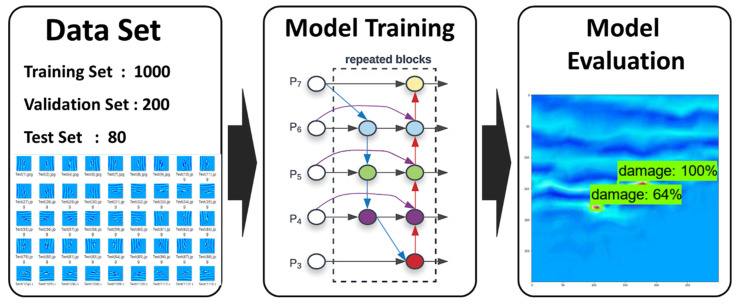
Experimental procedure of the CNN training.

In this study, to find out the applicability of the pipe damage detection model using the laser scanning data of the curved pipe part, dataset construction, data learning and detection, and evaluation were performed in three steps as shown in Figure 10.

In the first step, an ultrasound image of the pipe was acquired using a laser scanning technique, and an image dataset was constructed using it. In the second step, the CNN (EfficientDet) model was trained using the image dataset. Finally, the learned model was evaluated using the test set.

#### 4.2.1. Transfer Learning

**Figure 11:**
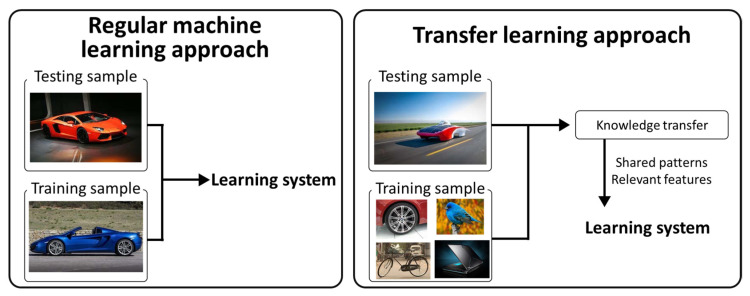
Basic frameworks of traditional machine learning approaches and knowledge transfer approaches [19].

The dataset used in this study comprises about 1280 images, and it is difficult to evaluate it with a general learning method. To this end, a transfer-learning-based EfficientDet model was applied using a COCO dataset [37] that was pretrained with about 330,000 images and 80 categories. The structure of the deep learning network is very complex, and as the amount of training data is small, problems such as overfitting occur and the learning performance deteriorates. As the amount of training data increases, the deep learning network performance improves [38]. In the field of image object detection, when it is difficult to collect specific data, such as an UWPI image used in this study, a transfer learning technique that learns new data using a model pretrained with a lot of data is a widely used technique in various deep learning applications [19,39]. The difference between the existing learning method and the transfer learning is shown in Figure 11. In this study, we train and evaluate the detection and evaluation of pipe bend damage by using the EfficientDet pretrained model using transfer learning.

#### 4.2.2. Train Dataset

In general, when developing deep learning algorithms, open image data that are freely available on the Internet such as ImageNet and COCO [37] are used a lot. However, in the case of open image data, the image is object-centered, and the background of the object is often simple and uncomplicated. However, open image data that can be used free of charge on the Internet did not have the UWPI images used in this study. Therefore, the images used in this study were acquired using laser scanning technology, which is detailed in Section 2 of this paper.

**Figure 12:**
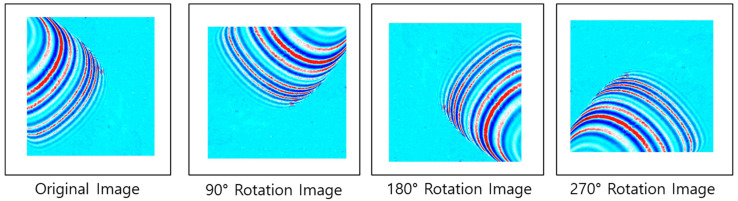
Laser scanning image dataset configuration.

The damage to the curved part of the pipe was scanned and the scan data were produced as UWPI image data using MATLAB software. The number of image data produced was 500, with size 1024 × 1024. Of the 500 scanned images, 320 pieces of data that can predict damage information were extracted. A total of 1280 training images were constructed by rotating by 90 degrees, as shown in Figure 12, for accurate deep learning construction.

To increase the resolution consistency and learning precision of the 1280 images, re-sizing was performed to a size of 512 × 512. The image data were divided into 1000, 200, and 80 images for the training, validation, and test sets, respectively. After dividing the image data set into training, validation, and test sets, the coordinate labeling work of the bounding box (the area of the actual damage location) was performed on each image using LabelImg software. In the case of the images used in this study, the class name was not designated, as damage was determined based on the laser scanning image of the pipe, and a collective label name “damage” was used.

#### 4.2.3. Training Dataset

The hardware specifications used in this study were: Intel Xeon^®^ Silver 4210 CPU, Nvidia GeForce RTX 3060, and 32GB RAM. The main software environment consisted of Anaconda, Python 3.8, TensorFlow 2.5.0, CUDA 11.2, Cudnn 8.1.1. The CNN-based pipe bend damage model was trained using the EfficientDet-d0 model [35].

The model was evaluated using intersection over union (IOU) and mean average precision (mAP), which are evaluation indicators that are often used in object detection. Unlike the existing object classification evaluation method, object detection requires both the evaluation of the class classification and the bounding box to find the position. In this study, since there is only one class (damage), the bounding box was evaluated.

**Figure 13:**
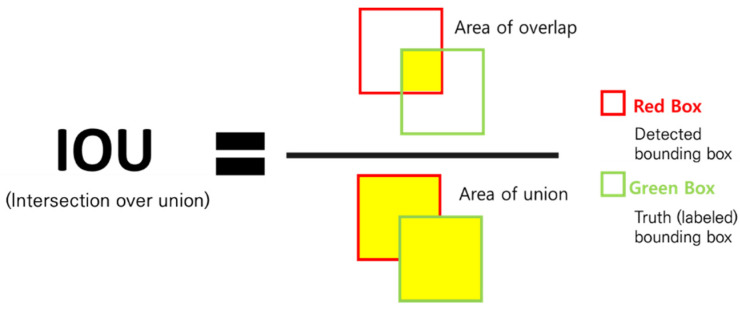
Intersection over union (IOU).

The calculation method of IOU is as shown in Figure 13, and it is an indicator of how well the bounding box is predicted. IOU indicates the ratio of the intersection of the bounding box labeled during the composition of the predicted area and the actual dataset to their union. In general, if the IOU value exceeds 0.5, it is judged as the correct answer [40].

Finally, the mAP is an index used as an evaluation criterion in PASCAL VOC, and it represents the performance of the object detection algorithm as an index, i.e., as an average value of average precision (AP) for each classification class [41]. Precision and recall are commonly used to evaluate the performance of detection models. Precision shows the ratio of detection of the true value to the total detection of data as in Equation (1), and recall refers to the ratio of detection of the true value to the cases of correct detection as in Equation (2). Since the two indicators are correlated with each other, AP, which is the area under the graph, is used in the precision–recall graph. The closer the AP value is to 1, the higher the performance of the object detection algorithm.

(1) $$\mathrm{Precision}=\frac{\mathrm{True}\;\mathrm{positive}}{\mathrm{True}\;\mathrm{positive}+\mathrm{False}\;\mathrm{positive}}$$

(2) $$\mathrm{Recall}=\frac{\mathrm{True}\;\mathrm{positive}}{\mathrm{True}\;\mathrm{positive}+\mathrm{false}\;\mathrm{negative}}$$

#### 4.2.4. UWPI Data Deep Learning Result

Prior to conducting this study, a transfer learning technique using a pretrained model used in object detection was applied to compensate for the lack of training data. Through the learning process, it was possible to know whether the used model was learning the image data well, by looking at the predicted values and the actual values. Learning was carried out in three stages as shown in Table 2. The same hardware specifications as well as the same batch size were applied for accurate comparison. For the batch size, step, and epoch values applied to training, Equation (3), which is widely used in the field of object detection, was used.

(3) Batch Size×Step=Epoch ×No. of samples

**Figure 14:**
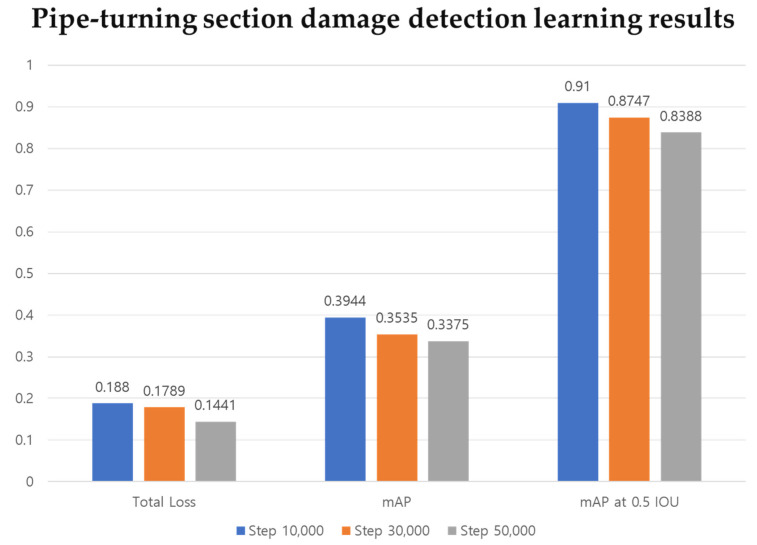
Comparison of deep learning results according to steps (Total loss, mAP, mAP at 0.5 IOU).

Figure 14 shows the learning results after 10,000, 30,000 and 50,000 steps. The sum of damage detection loss and bounding box regression loss for learning according to each step is summarized as total loss. From the results of a total of three learning stages, it was confirmed that the total loss was less than 0.2. Comparing results after 10,000 steps and 50,000 steps, the loss decreases as repeated learning progresses to 0.188 and 0.1441, respectively. In addition, the learning progresses normally.

As a result of performance evaluation for the trained model, the average mAP values of the pipe damage data learning were calculated as 0.3944, 0.3535, and 0.3375, (as shown in Figure 13) and the average mAP values at 0.5 IOU were calculated as 0.91, 0.8747, and 0.8388, after 10,000, 30,000, and 50,000 steps, respectively. Observing that the average mAP value of the COCO 2017 pretrained CNN (EfficientDet-d0) algorithm used in this study was 0.336 [35], it can be deduced that the learning proceeded normally. The evaluation was conducted using a preclassified test image data set before the learning. As a result of evaluating a total of 80 test images as evaluation data, the results shown in Table 3 below were obtained.

**Figure 15:**
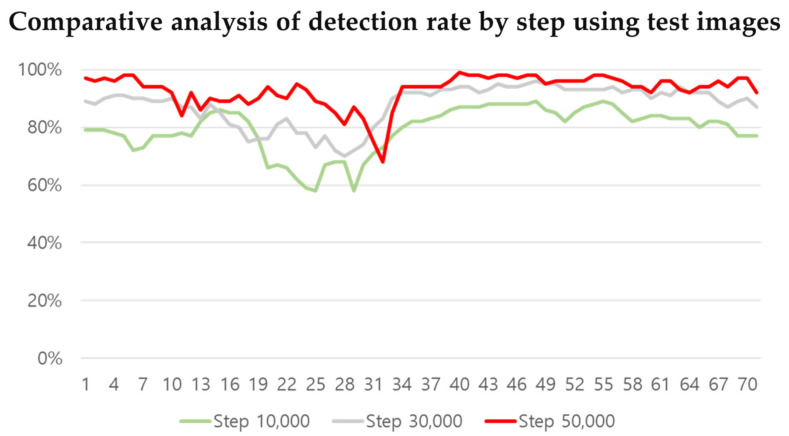
Comparative analysis of detection rate by step using test images.

Following the evaluation at the 10,000, 30,000, and 50,000 step, average detection rates of 75%, 86%, and 88%, respectively, were confirmed. When evaluating the performance of the learning model, the average mAP was lower at steps 30,000 and 50,000 compared to step 10,000. However, because of the direct evaluation, the damage detection rate was higher at step 50,000. At step 10,000, the detection rate ranged from 50% to 89%, resulting in an average detection rate of 75%. At step 30,000 it ranged from 53% to 96%, and at step 50,000 it ranged from 50% to 99%. To see the overall aspect of learning, the undetected data are excluded and are shown in a graph in Figure 15. Taking a close look at the graph, it can be seen that the most accurate result was obtained after 50,000 steps.

**Figure 16:**
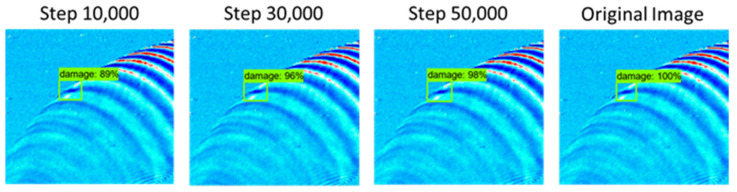
Test result of damage detection with excellent accuracy.

**Figure 17:**
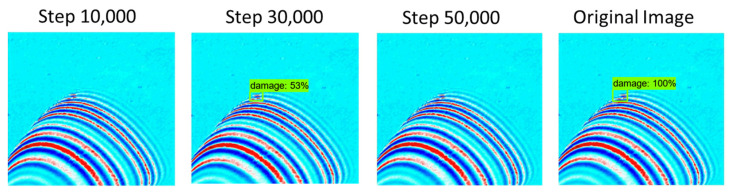
Test result of damage not detected and low accuracy.

Figure 16 shows the test result with the highest detection rate compared to the original image data and it can be seen that an average detection rate of 89% or more was achieved compared to the original image data. Observing the overall test, no erroneous detection occurred. However, at steps 10,000 and 50,000, three non-detections occurred as shown in Figure 17.

In general, the main cause of non-detection in learning results is that there is no difference in color or contrast between an object and the background. This problem is due to a shape that appears depending on the background and physical environment such as color or lighting of the object [42].

From the results of the test, no erroneous detection was found in this study, and three cases of non-detection occurred at steps 10,000 and 50,000. This is thought to be for the following reasons. First, regarding the undetected results, the problem is that there is no difference in contrast between the background color of the image and the color of the damaged part, which is believed to have affected the learning results. Second, it is presumed that some non-detection occurred because there was no experience in learning the UWPI image of this study with the COCO 2017dataset. Therefore, it can be deduced that it will be improved if many pipe UWPI images are acquired and used with deep learning in order to improve detection.

## 5. Conclusions

In this study, we proposed an automatic damage detection system for pipe bends using a CNN object detection algorithm with laser scanning data to efficiently extend the safety management of pipes used in the construction industry and many industries. Using a Q-switched Nd:YAG pulse laser and an acoustic emission (AE) sensor, UWPI image data were produced for the detection of damage introduced artificially to the pipe bend. A damage detection system was constructed using a total of 1280 training images obtained through post-processing of the UWPI data. Since 1280 images are insufficient to proceed with deep learning, a transfer learning technique using the pretrained COCO 2017 EfficientDet-d0 algorithm was applied.

Examining the learning model using the pipe damage data, it was confirmed that the detection performance index, mAP, was higher than the value of 0.336 from the COCO 2107 Effi-cientDetd-0 model. This indicates that the model training was successful, and it was confirmed that there was no performance difference when comparing the existing methods of learning that use a lot of data with the one implemented through transfer learning with 1280 pieces of data. From the result of the CNN learning using pipe damage data, three cases were not detected after 10,000 steps and 50,000 steps. It was deduced that a small amount of non-detection occurred due to an insufficient quality and quantity of images. Therefore, to supplement the undetected problem, we intend to proceed with the following additional research.

- Through additional experiments and research, we intend to secure UWPI data according to the damage size using laser scanning techniques for the components (curved part, curved pipe part, bolted joint part, welding, etc.) of pipes.
- This study confirmed the possibility of detecting damage to pipes based on laser scanning through the transfer learning technique, and based on this, we intend to propose a better detection technique using new algorithms and large amounts of data.
- To acquire ultrasonic signals in the laser scanning system, this study used the AE sensor installed directly on the pipe. Therefore, we intend to develop a noncontact nondestructive system for efficient pipe damage detection by using laser diameter vibration (LDV) instead of an AE sensor.

In this study, using the UWPI system and CNN, an automatic pipe bend damage detection system was proposed. Therefore, it is expected that efficient maintenance will be possible for piping used in construction and many industries.

## Figures and Tables

**Table 1:** Specifications of the laser system.

Laser Head: Brilliant Ultra GRM100	Galvanometer: Scancube 10
Wavelength: 1064 nm	Wavelength: 1064 nm
Energy per pulse: 100 mJ	Tracking error: 0.16 ms
Pulse repetition rate: 20 Hz	Positioning speed: 10 m/s
Pulse duration: 6.5 ns	Max. angular velocity: 100 rad/s
Beam diameter: 3 mm	(within 0.35 rad)

**Table 2:** Pipe damage detection CNN training configuration information.

Batch Size	Steps	Epochs	No. of Samples
8	10,000	80	1000
8	30,000	240	1000
8	50,000	400	1000

**Table 3:** Damage detection rate of test images for each step.

Test Image	Step 10,000	Step 30,000	Step 50,000
1	79%	89%	97%
2	79%	88%	96%
3	79%	90%	97%
4	78%	91%	96%
5	77%	91%	98%
6	72%	90%	98%
7	73%	90%	94%
8	77%	89%	94%
9	77%	89%	94%
10	77%	90%	92%
11	78%	87%	84%
12	77%	87%	92%
13	82%	83%	86%
14	85%	88%	90%
15	86%	85%	89%
16	85%	81%	89%
17	85%	80%	91%
18	82%	75%	88%
19	76%	76%	90%
20	66%	76%	94%
21	67%	81%	91%
22	66%	83%	90%
23	62%	78%	95%
24	59%	78%	93%
25	58%	73%	89%
26	67%	77%	88%
27	68%	72%	85%
28	68%	70%	81%
29	58%	72%	87%
30	67%	74%	83%
31	71%	80%	75%
32	73%	83%	68%
33	77%	90%	85%
34	80%	92%	94%
35	82%	92%	94%
36	82%	92%	94%
37	83%	91%	94%
38	84%	93%	94%
39	86%	93%	96%
40	87%	94%	99%
41	87%	94%	98%
42	87%	92%	98%
43	88%	93%	97%
44	88%	95%	98%
45	88%	94%	98%
46	88%	94%	97%
47	88%	95%	98%
48	89%	96%	98%
49	86%	95%	95%
50	85%	95%	96%
51	82%	93%	96%
52	85%	93%	96%
53	87%	93%	96%
54	88%	93%	98%
55	89%	93%	98%
56	88%	94%	97%
57	85%	92%	96%
58	82%	93%	94%
59	83%	93%	94%
60	84%	90%	92%
61	84%	92%	96%
62	83%	91%	96%
63	83%	94%	93%
64	83%	92%	92%
65	80%	92%	94%
66	82%	92%	94%
67	82%	89%	96%
68	81%	87%	94%
69	77%	89%	97%
70	77%	90%	97%
71	77%	87%	92%
72	63%	68%	64%
73	51%	57%	0%
74	0%	66%	52%
75	58%	69%	0%
76	50%	70%	53%
77	0%	53%	0%
78	0%	56%	58%
79	67%	76%	50%
80	87%	93%	98%
Average detection rate	75%	86%	88%
